# MUC5B Leads to Aggressive Behavior of Breast Cancer MCF7 Cells

**DOI:** 10.1371/journal.pone.0046699

**Published:** 2012-10-02

**Authors:** Hélène Valque, Valérie Gouyer, Frédéric Gottrand, Jean-Luc Desseyn

**Affiliations:** 1 Inserm U995, Lille, France; 2 University Lille 2, Lille, France; 3 CHRU of Lille, Lille, France; Wayne State University School of Medicine, United States of America

## Abstract

The mucin MUC5B has a critical protective function in the normal lung, salivary glands, esophagus, and gallbladder, and has been reported to be aberrantly expressed in breast cancer, the second leading cause of cancer-related deaths among women worldwide. To understand better the implication of MUC5B in cancer pathogenesis, the luminal human breast cancer cell line MCF7 was transfected with a vector encoding a recombinant mini-mucin MUC5B and was then infected with a virus to deliver a short hairpin RNA to knock down the mini-mucin. The proliferative and invasive properties in Matrigel of MCF7 subclones and subpopulations were evaluated *in vitro*. A xenograft model was established by subcutaneous inoculation of MCF7 clones and subpopulations in SCID mice. Tumor growth was measured, and the tumors and metastases were assessed by histological and immunological analysis. The mini-mucin MUC5B promoted MCF7 cell proliferation and invasion *in vitro*. The xenograft experiments demonstrated that the mini-mucin promoted tumor growth and MCF7 cell dissemination. In conclusion, MUC5B expression is associated with aggressive behavior of MCF7 breast cancer cells. This study suggests that MUC5B may represent a good target for slowing tumor growth and metastasis.

## Introduction

Mucins are high molecular weight *O*-glycosylated proteins present at the surface of most epithelial cells. Human mucins are classified structurally into two families: membrane-bound mucins (MUC1, MUC3A/B, MUC4, MUC12, MUC13, MUC16, and MUC17) and secreted or gel-forming/polymerizing mucins (MUC2, MUC5AC, MUC5B, MUC6, and MUC19) [1]–[3]. Secreted mucins comprise amino- and carboxy-terminal regions that share similar domains with the pro-von Willebrand factor. These mucins polymerize through these regions to form long polymers, which are secreted into the lumen of many organs. The central part of mucins contains large regions enriched in hydroxyamino acids and proline (Ser/Thr/Pro regions) that are substituted extensively with *O*-glycans. These Ser/Thr/Pro sequences differ in size and in sequence between mucins, are not conserved between species, and are usually organized in tandem repeats (TR). In many polymerizing mucins, these tandemly repeated sequences are linked to or interrupted by a naked and hydrophobic domain rich in cysteine residues and called a CYS domain [4].

We have extensively characterized the human *MUC5B* gene structure [5]–[7]. The central part of MUC5B is composed of three alternating subdomains: (i) a subdomain named R domain, found five times and made of repetitions of an irregular repeat of 29 amino acids (aa) rich in Ser, Thr, and Pro; (ii) a conserved subdomain of 111 aa called the R-end domain and found four times; and (iii) the highly conserved domain named the CYS domain [4], which is about 110 aa long and found seven times in MUC5B protein. The alternating CYS domain/R domain/R-end creates a larger composite repeat unit of 528 aa [6].

Breast cancer is the second leading cause of cancer-related deaths in women worldwide [8]. The membrane bound mucin MUC1 is the most investigated mucin in breast cancer [9] and is the most widely studied mucin for developing therapy to treat breast cancer [8]. Among the four polymerizing mucins *MUC2*, *MUC5B*, *MUC5AC*, and *MUC6* whose altered expression has been reported in breast cancer tissues, the role of MUC5B is poorly documented. Only a few studies have focused on MUC5B. The mucin was detected by immunohistochemistry in primary breast tumors (81%) and in samples of normal-appearing breast epithelia adjacent to cancer cells (42.1%), whereas MUC5B was not detected in normal control breast samples [10]. *MUC5B* mRNA transcripts were detected in bone marrow aspirates of 9/46 patients (19.5%) who underwent primary tumor resection [11] but not in 36 samples of normal peripheral blood samples, suggesting that MUC5B may be a specific marker with a high specificity (100%) for the diagnosis of breast cancer cell dissemination [10], [11]. To analyze the role of MUC5B in breast tumorigenesis, we transfected the MCF7 luminal breast tumor cell line [12] with a plasmid encoding a mini-MUC5B mucin made of large composite unit of MUC5B with many *O*-glycosylated sites and flanked by two CYS domains. We found that the mini-mucin MUC5B promotes cell proliferation and *in vitro* invasion of tumor breast cancer cells. Using a xenograft immunodeficient mouse model, we show that MUC5B promotes tumor growth and metastasis. These data suggest that MUC5B represents a good therapeutic target for slowing tumor growth and dissemination of breast cancer.

**Figure 1:**
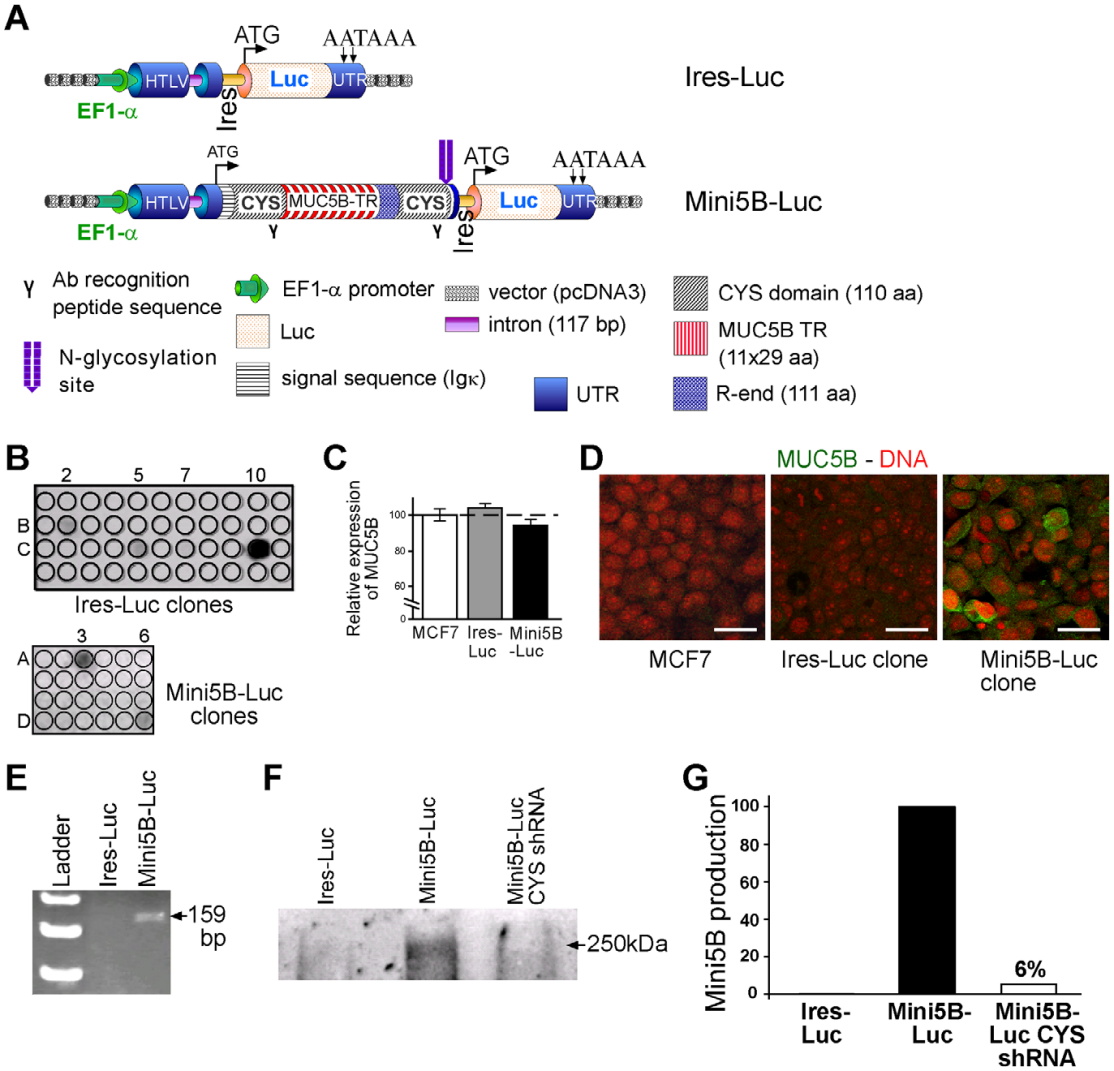
Generation of stable clones expressing or not expressing the mini-mucin Mini5B. (**A**) Schematic representation of the control Ires–Luc and Mini5B–Luc vectors. The insert encoding the Mini5B was made by a signal sequence, 11 TR of 29 aa, one R-end domain of 111 aa, and two CYS domains of 110 aa. (**B**) Using the luciferase activity assay, four stable clones expressing the Ires–Luc transgene (clones B2, C5, C7, and C10) and two stable clones expressing the Mini5B–Luc transgene (clones A3 and D6) were obtained. (**C**) *MUC5B* mRNA expression was studied by qRT–PCR (TaqMan) in MCF7 parental cell line (white box), in MCF7 Ires-Luc clone (grey box) and in Mini5B-Luc clone (black box). Expression levels were normalized to mRNA levels of 18S and shown as x-fold relative to the normalized expression of *MUC5B* gene in MCF7 parental cells. Relative amounts of target genes were calculated using the ΔΔCt method. Values are means ± standard deviation from four to six independent samples. (**D**) MUC5B immunofluorescence analysis of MCF7 parental cells, Ires–Luc and Mini5B–Luc clones. Mini5B was secreted at the cell surface of Mini5B–Luc clones. Nuclei were counterstained with propidium iodide. Scale bar 50 µm. (**E**) Expression of the Mini5B was evaluated by RT–PCR. A PCR product of 159 bp was detected only in the Mini5B–Luc clone. (**F**) Western blot analysis of lysates from Ires–Luc clones, Mini5B–Luc clones, and Mini5B–Luc CYS shRNA subpopulation. (**G**) Inhibition of Mini5B production detected by Western blotting was quantified. Mini5B production of the clone Mini5B–Luc was set at 100 and the production of Ires–Luc was set at 0.

## Materials and Methods

### Cell culture

The human breast cancer cell line MCF7 was purchased from American Type Culture Collection (ATCC HTB22; derived from a human breast adenocarcinoma). Cells were maintained in minimal essential medium (MEM) (Invitrogen/Life technologies, Villebon-sur-Yvette, France) supplemented with 2 mM l-glutamine, 1.5 g/L sodium bicarbonate, 0.1 mM nonessential aa, 1 mM pyruvate sodium, 0.01 mg/mL bovine insulin, and 10% fetal bovine serum (Thermo Scientific) at 37°C in a humidified atmosphere of 5% CO_2_.

**Figure 2:**
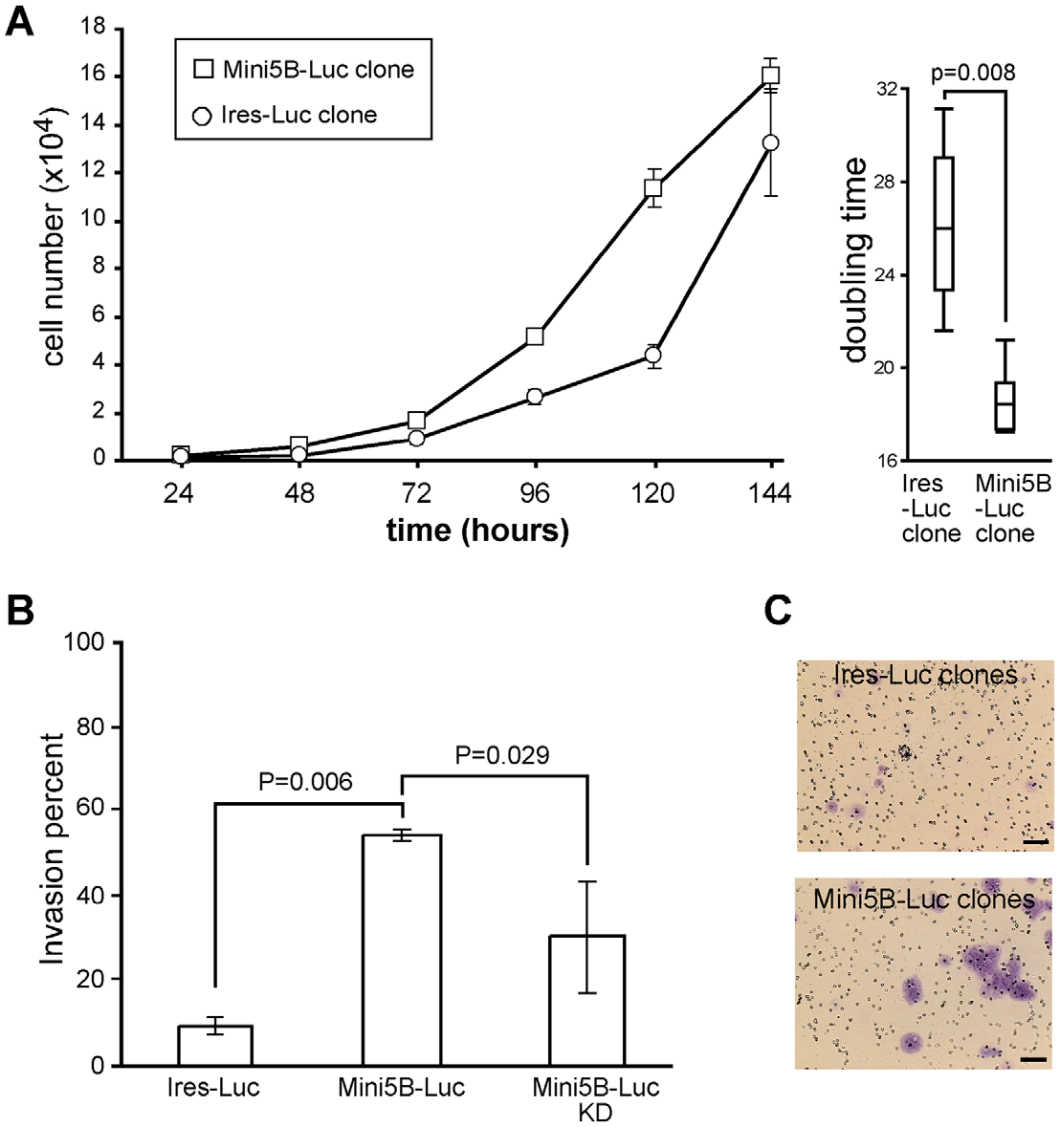
Promotion of proliferation and invasion of MCF7 cells *in vitro* by Mini5B. (**A**) Growth curves of Mini5B–Luc and Ires–Luc clones. Data are expressed as the mean ± SEM. The curve is representative of three experiments. Doubling time was calculated for the two clones. (**B**) Invasion percentages of Ires–Luc and Mini5B–Luc clones and Mini5B–Luc KD population. Data are expressed as mean ± SEM. The diagram is representative of two experiments. (**C**) Illustration of invading cells stained with toluidine blue. Scale bar 50 µm.

### Mini5B expression vector

An IRES–Luc (noted thereafter Ires-Luc) cassette flanked by two *Eco*RI restriction sites was generated by a two-step PCR amplification and subcloned into the pCR4 vector (Invitrogen) using pMG (InvivoGen, Toulouse, France) and the pGL3 Basic (Promega, Leiden, The Netherlands) as the templates. The 1.7 kb Luc sequence was amplified using the two oligonucleotide sequences 5′-AGATCTTACGCGTGCTAGCC-3′ (forward) and 5′-GGATCCGACTCTAGAATTACAC-3′ (reverse) introducing a *Bg*lII and a *Bam*HI restriction site, respectively (underlined). The 0.7 kb Ires sequence was amplified using the two oligonucleotide sequences 5′-GAATTCGAACGTAGCTCTAG-3′ (forward) and 5′-AGATCTCCTACCGGTGATCTC-3′ (reverse) introducing an *Eco*RI and a *Bgl*II restriction site, respectively (underlined). The empty eukaryotic-expressed vector pcMG and the pcMG carrying a mini5B sequence were used [13]. The plasmid pcMG is a derivative of the pcDNA3.1 plasmid but the promoter has been replaced with the EF1α-HTLV composite promoter from pMG and contains a small intron. The Mini5B sequence is made of the IgK leader chain in frame with two identical CYS domains of MUC5B flanking a large Ser/Thr/Pro region (430 aa) of the mucin. The 2.5 kb *Eco*RI–*Eco*RI–Ires–Luc cassette was subcloned into the unique *Eco*RI restriction site of the pcMG or into the pcMG–Mini5B, just downstream of the stop codon of Mini5B. Only vectors with the correct orientation of the cassette (pcMG–Ires–Luc and pcMG–Mini5B–Ires–Luc) were used. Mini5B contains in its carboxy-terminal sequence the *N*-glycosylation site NSS.

**Figure 3:**
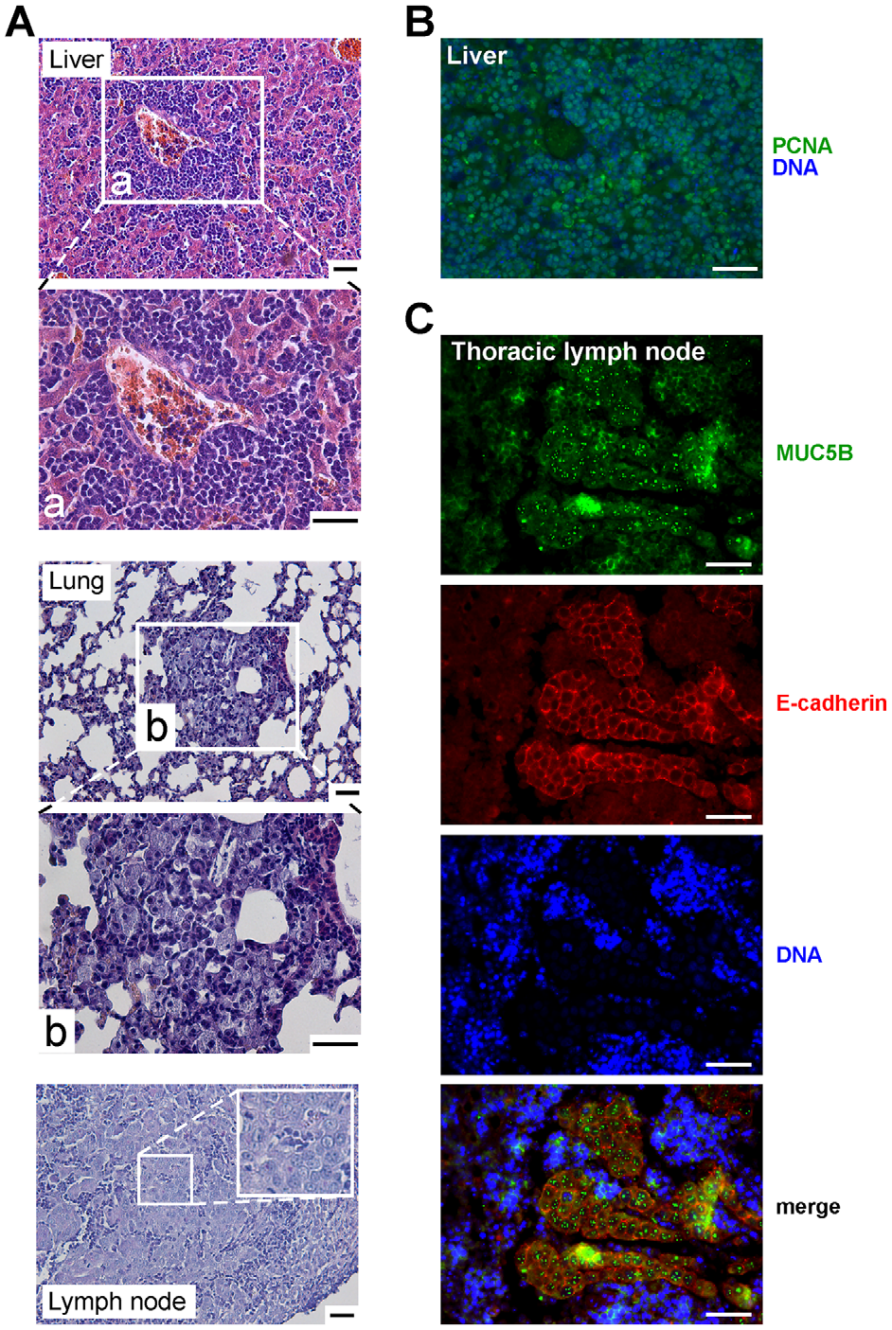
Histological analysis of mice sacrificed during the experimentation. (**A**) HE staining was performed on paraffin-embedded sections of the liver, lungs, and lymph nodes of mice injected with Mini5B–Luc clones. Metastasis is showed in the magnified image. (**B**) Immunofluorescence analysis was performed on paraffin-embedded sections of liver from mice injected with Mini5B–Luc clones and stained with anti-PCNA antibody. Metastatic cells were PCNA positive. (**C**) Double immunofluorescence analysis using the anti-human E-cadherin and anti-MUC5B antibodies were performed on paraffin-embedded sections of thoracic lymph nodes from mice injected with Mini5B–Luc clones. Metastatic cells expressed both the epithelial marker E-cadherin and secreted MUC5B protein. Nuclei were counterstained with Hoechst 33258 or propidium iodide. Mt, metastasis. Scale bar 50 µm.

### Stable transfection and selection of cells

MCF7 cells were transfected using the Effectene kit (Qiagen, Courtaboeuf, France) with 2 µg of the pcMG–Mini5B–Ires–Luc or the pcMG–Ires–Luc (Luc clones) plasmid linearized with the unique *Ssp*I restriction enzyme. Transfected cells were then selected with the neomycin analogue G418 (650 µg/mL; Invitrogen) for 10 days through the neomycin-resistance gene of the pcMG vector. G418-resistant clones were selected individually and seeded in a 96-well culture plate, expanded in a 48-well plate then a six-well plate, and finally in a 25 cm^2^ culture flask.

**Figure 4:**
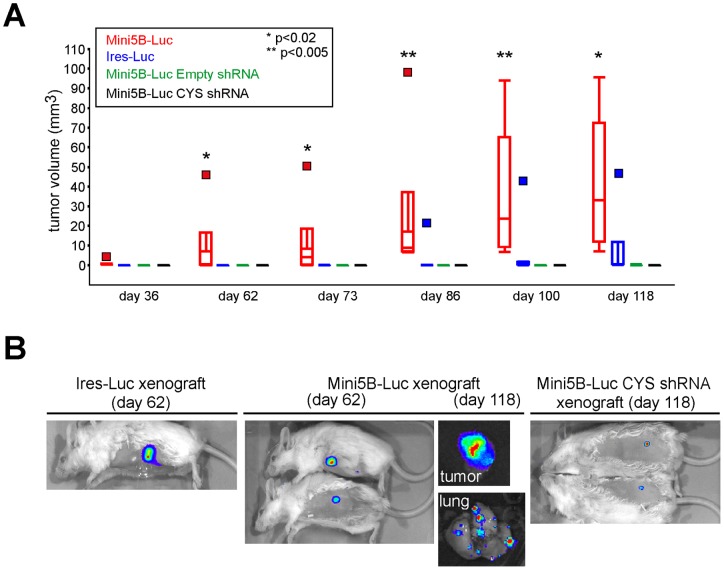
Impact of Mini5B expression on the growth of MCF7 cell xenografts in immunodeficient mice. (**A**) Mini5B–Luc, Ires–Luc, Mini5B–Luc empty shRNA, or Mini5B–Luc CYS shRNA xenografts. Tumor growth was monitored once a week for 118 days. *P<0.02, ** *P*<0.005. (**B**) Examples of tumor growth and metastasis on days 62 and 118 in anesthetized mice using the Xenogen apparatus.

### Luciferase activity assay

A luciferase activity assay was used to isolate clones expressing the transgene. Cells were incubated for 5 min with 70 µL of Luc substrate (Superlight Luciferase Reporter gene assay, Gentaur, Paris, France). The bioluminescence of cells was measured using a CCD camera (LAS 3000, Fujifilm). Luciferase activity was expressed in arbitrary unit/mm^2^ by the Multi Gauge Fujifilm software.

**Figure 5:**
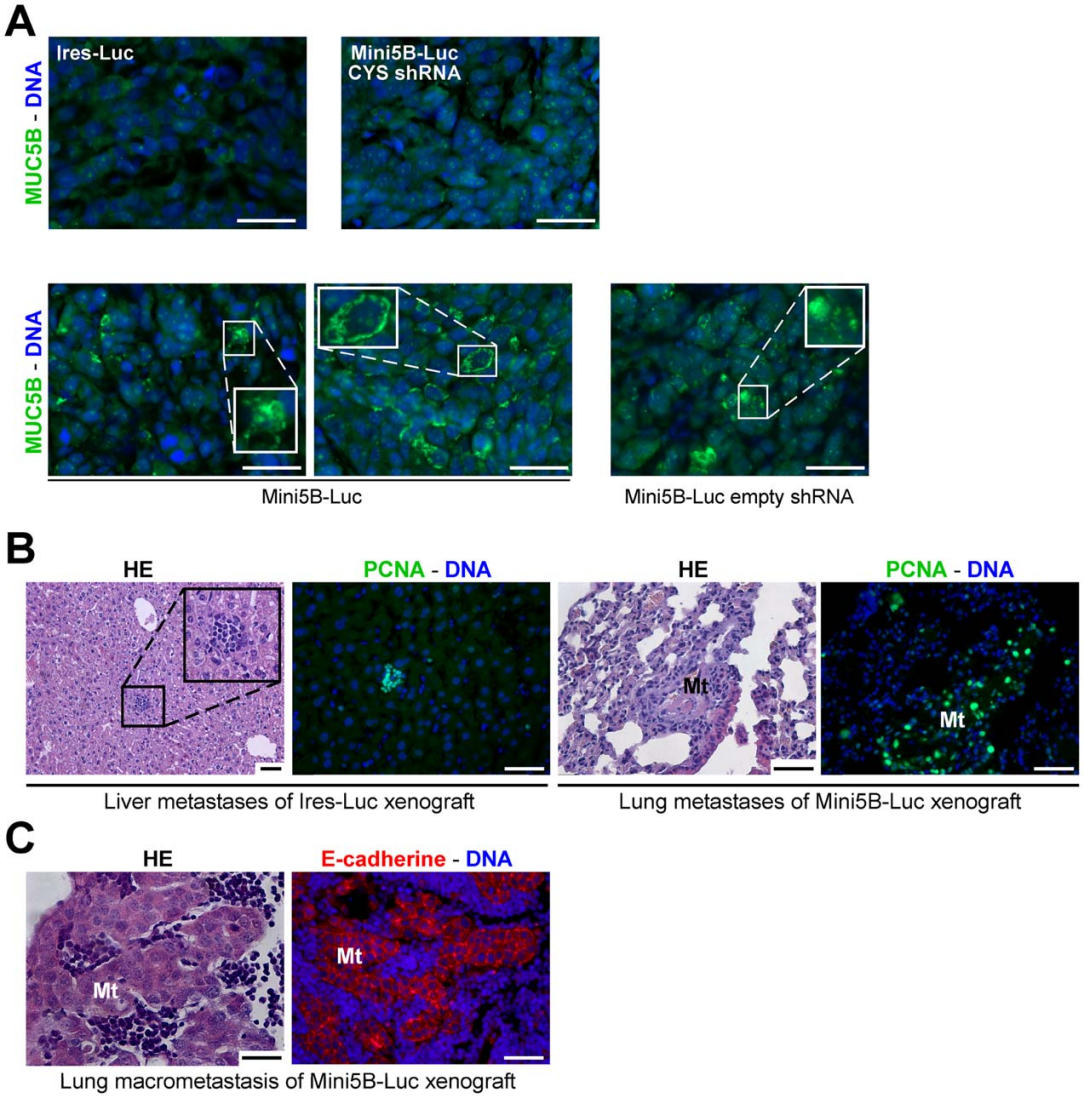
Impact of Mini5B expression on the dissemination of MCF7 cell xenografts in immunodeficient mice. (**A**) Immunofluorescence analysis was performed on paraffin-embedded sections of tumors from mice injected with the Ires-Luc clone, the Mini5B-Luc CYS shRNA clone, the Mini5B-Luc clone and the Mini5B-Luc empty shRNA clone and stained with anti-MUC5B antibody. Mini5B was expressed and secreted by tumoral cells in tumors of Mini5B-Luc group and Mini5B-Luc empty shRNA. (**B**) Serial sections of livers of mice injected with the Ires–Luc clone and stained with HE and anti-PCNA antibody, and serial sections of lungs of mice injected with the Mini5B–Luc clone and stained with HE and anti-PCNA antibody. Metastases in the liver and the lung were visualized. Metastatic cells were PCNA positive. (**C**) Histological analysis of a macrometastasis in lung of a mouse injected with the Mini5B–Luc clone using HE staining and E-cadherin immunostaining (serial sections). Mt; metastasis. Nuclei were counterstained with Hoechst 33258. Scale bar 50 µm.

### Inhibition of Mini5B expression by retroviral-mediated siRNA

Mini5B-knock down (KD) MCF7 cells were generated using a retrovirus-mediated RNA interference system as described previously [14]. In brief, oligonucleotides encoding short hairpin RNAs (shRNAs) directed against CYS#4 sequence mRNA were designed according to recommendations described in Schuck et al. [14]. The RNA interference hairpin was designed to target the CYS#4 sequence: 5′-GTCTGCAGGAACCGTGAGCAG-3′. Cells (0.4×10^6^) were seeded into a six-well plate. On the next day, cells were infected with either a control virus (RVH1 without a hairpin insert) or a CYS#4-KD-virus in the presence of 8 µg/mL polybrene (hexadimethrine bromide; Fluka/Sigma Aldrich, Saint-Quentin Fallavier, France). Cells were incubated at 37°C overnight, and the infection was repeated twice with a fresh batch of virus. One day after the last infection, transduced cells were selected in the presence of 1.2 µg/mL puromycin for three days. One week after selection, the KD efficiency was analyzed by luciferase activity assay. The percent inhibition was calculated from the formula [(luciferase activity CYS shRNA)/(luciferase activity empty shRNA)]×100.

**Figure 6:**
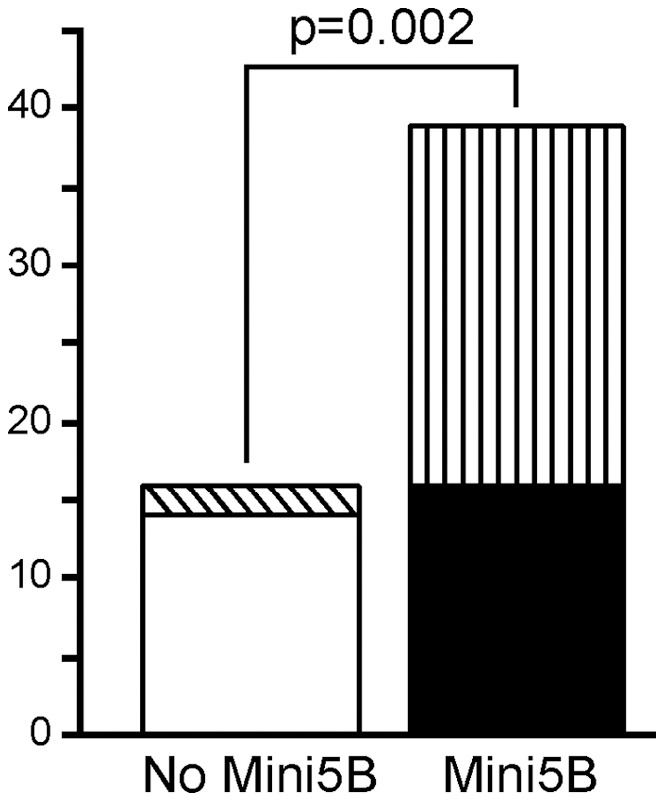
Clinical scores of xenografted SCID mice. The empty box and the box with the diagonal lines are for the Ires–Luc clone and the Mini5B KD groups, respectively. The black box and the box with vertical lines are for the Mini5B–Ires empty shRNA and the Mini5B–Ires–Luc groups, respectively.

### Reverse transcription-polymerase chain reaction (RT-PCR)

The transgene expression was detected by RT-PCR. Total RNA was extracted from cells using TRIzol reagent (MRC/Euromedex, Souffelweyersheim, France) as recommended by the manufacturer. Total RNA (2 µg) was reverse transcribed using the Advantage RT kit for PCR (Clontech) according to the manufacturer's protocol. PCR was performed on 5 µL of cDNA using the two specific primers: 5′-GGAGCCTACCTAGACTCAGCC-3′ (forward) and 5′-CCCATAGCAGTGTGTCTGTC-3′ (reverse) complementary to a sequence of the HTLV genomic sequence and to the CYS sequence, respectively. The expected size is 159 bp. G3PDH was amplified as an internal control.

**Table 1:** Metastases following subcutaneous xenograft.

	Mice	Presence of metastases			
Groups	injected^a^	on day 118			Macrometastasis^b^
		Liver	Lung	Lymph nodes	on day 118
Ires–Luc	6	2/5┐	2/5┐	2/5	1/5 (lung)
Mini5B–Ires–Luc CYS shRNA	6	1/6┘┐	0/6┘┐	0/6	0/6
Mini5B–Ires–Luc empty shRNA	4	3/4┐┘*	2/4┐┘*	1/4	0/4
Mini5B–Ires–Luc	6	4/4┘	4/4┘	3/4	4/4 (lung); 1/4 (liver)

a mice were injected with 10×10^6^ cells.

b the presence of macrometastases was evaluated by histological analysis.

* *P* = 0.02. Mice injected with MCF7 clones/population expressing Mini5B were grouped and mice injected with clones/cell population not expressing Mini5B were grouped.

### Quantitative RT-PCR (qRT-PCR) of *MUC5B*

For quantitative PCR analysis of *MUC5B* expression, cells were washed and harvested into sterile PBS, pelleted by centrifugation and rapidly frozen in liquid nitrogen and stored at −80°C until RNA extraction. Total RNA extraction, cDNA synthesis and PCR experiments using 18s as internal positive control were performed as previously described [15]. Primer and TaqMan probe sequences were selected using the Primer3 freeware within the 3′-end of human *MUC5B* cDNA. The specific primers and probe for *MUC5B* were as follows: forward primer 5′-GCCTGCTGCAGGGTAACTCA-3′, reverse primer 5′-ATTGCTCAGGGTTTATTTGCAAA-3′ and probe 5′-CATCCCAAAGCCCCCTCTGCTCA-3′. All samples were measured in triplicate. The cycle threshold values of all samples were measured by the ABI Prism 7700 sequence detector system (Applied Biosystems) and MUC5B expression levels were normalized to mRNA levels of 18S ribosomal RNA. Relative amounts of target genes were calculated using the ΔΔCt method.

### Confocal microscopy

For confocal microscopy, cells grown on plastic glass coverslips were fixed with 4% paraformaldehyde for 20 min. The fixation was stopped with 50 mM NH_4_Cl in PBS for 20 min. Cells were permeabilized in 0.2% saponin for 20 min and incubated in blocking reagent (PBS, 1% bovine serum albumin, 0.2% saponin) for 30 min. Anti-MUC5B (1∶200) [16] antibody diluted in blocking reagent was added overnight at 4°C. After incubation with the primary antibody, the cells were rinsed three times with PBS for 5 min and incubated with fluorescein isothiocyanate (FITC)-conjugated secondary antibody (Jackson ImmunoResearch Laboratories, West Grove, PA) (1∶150) diluted in blocking reagent for 2 h. Nuclei were counterstained with propidium iodide solution (1∶1000) for 5 min. The immunolabeled sections were dried and mounted with Mowiol mounting medium and stored at 4°C. Images were acquired using confocal microscopy (Leica TCS LCSM, Leica Microsystems, Exton, PA) and were processed identically and minimally by importing them into Adobe Photoshop CS software (Adobe Systems, Inc., Mountain View, CA).

### Protein analysis

Cells were washed five times with PBS, scraped and centrifuged at 1300×*g* for 5 min, and the pellet was resuspended in 200 µL PBS containing 0.2 mM 4-(2-aminoethyl) benzenesulfonyl fluoride hydrochloride. After freeze–thawing, cells were sonicated, and the protein concentration was measured using the BCA protein assay (Pierce Biotechnology, Rockford, IL). Protein lysates (150 µg) were loaded onto an 8% SDS-polyacrylamide gel under reducing conditions and transferred to a Hybond-C extra membrane (Hybond ECL Amersham Bioscience/GE Healthcare, Velizy-Villacoublay, France). The membrane was blocked with 5% powered milk in PBS/0.1% Tween 20 overnight, washed, and probed with the anti-MUC5B antibody [16] diluted at 1∶400 in PBS/0.1% Tween for 3 h. After washing, the membrane was incubated for 45 min with horseradish peroxidase-conjugated goat anti-mouse antibody (Santa Cruz Biotechnologies, Heidelberg, Germany) diluted at 1∶4000 in PBS/0.1% Tween. Detection was performed by luminescence using the ECL Western Blotting System (Amersham Biosciences/GE Healthcare). Inhibition of Mini5B expression at the protein level was analyzed by Western blotting using the Image Station 4000 MM PRO (Carestream Health Inc., Woodbridge, CT) and quantified using Quantity One (V4.2.3, Bio-Rad, Marnes La Coquette, France) software.

### Cell proliferation

Mini5B–Luc and the control Ires–Luc clones were plated at 2×l0^3^ cells/well in 96-well tissue culture plates. Every 24 hours, three replicative wells were trypsinized, stained with trypan blue (0.05%) and the vital cells were counted in a Malassez counting chamber. A growth curve (cell number) was established. Data are expressed as mean ± SEM. The population-doubling time was calculated from its exponential growth phase (from 72 hours to 120 hours).

### Cell invasion assay

The *in vitro* invasive properties of MCF7 cells were studied using Matrigel inserts (BD Biocoat, BD Biosciences, Le Pont de Claix, France). Serum-deprived cells (5×10^4^ cells) were loaded in the upper compartment of the chambers. After incubation for 24 h, the membranes were processed and the invading cells were counted in 10 random fields under a microscope. The invasion percentage was expressed as the percentage of invading cells through the Matrigel relative to the percentage of migrating cells through the control inserts. A representative graph of three independent experiments is reported. Data are expressed as mean ± SEM.

### Tumorigenicity assay

Subconfluent MCF7 cells were trypsinized, centrifuged at 1300×*g* for 5 min, washed twice with MEM without serum and antibiotics, and resuspended in serum-free cold medium. A total volume of 0.2 mL containing 10×10^6^ cells was injected subcutaneously (s.c.) into the flank of 17-week-old female immunodeficient SCID Beige mice (CB17.Cg-Prkdc-Lyst/Crl, Charles River, France); six mice were used per group except for the Mini5B–Luc KD RVH1 group, where four mice were injected. This experiment was performed with Mini5B–Luc, Mini5B–Luc RVH1 KD, Mini5B–Luc CYS KD, and Ires–Luc cells. The tumor dimensions were measured once a week, and the volume (V, mm^3^) was calculated as V = 0.5236×L×W^2^, where L and W are the length and width of the tumor xenografts, respectively. The tumor growth and metastasis appearance were also evaluated by measuring luminescence using the IVIS50 apparatus (Xenogen, Caliper Life Sciences, Villepinte, France). Ten minutes before the measurement of luminescence, mice were injected intraperitoneally with luciferin (30 mg/kg) and then anesthetized with ketamine (100 mg/kg) and xylazine (10 mg/kg) and kept at 37°C.

### Ethics Statement

The animal procedure followed in this study had been approved by the Animal Care Ethics Committee of the region Nord-Pas-de-Calais (protocol CEEA 142011). The animal care and all procedures were in accordance with the French Guide for the Care and Use of Laboratory Animals and with the guidelines of the European Union.

### Removal, fixation of tissues, and staining

Mice were sacrificed by a lethal injection of xylazine and ketamine. The tumors, lungs, spleens, and livers were harvested, inspected macroscopically, fixed in 4% paraformaldehyde, processed, embedded, sectioned, stained with hematoxylin and eosin (HE), and analyzed for the presence of tumors and metastases.

### Immunohistochemistry

Immunohistochemistry of paraffin sections was performed as described [17]. Anti-MUC5B (1∶250; [18]), anti-E-cadherin (Abcam HECD1, 1∶400), and anti-proliferating cell nuclear antigen (PCNA) (Abcam, ab29; 1∶1,000) antibodies were used together with FITC-conjugated anti-rabbit-IgG goat antibody (1∶150). For PCNA, E-cadherin and MUC5B immunostaining, the sections were pretreated by immersion in sodium citrate buffer (10 mM sodium citrate, 0.05% Tween, pH 6.0) at 95–100°C for 20 min and then at room temperature for 20 min. Slides were incubated with Hoechst 33258 (1∶1,000; Molecular Probes Inc., Life Technologies, Saint Aubin, France) solution for 5 min for nuclei counterstaining. Images were acquired as described [19].

### Clinical score

A post hoc clinical score was determined by scoring the number of dead mice before the end of the experimentation (3 points); the presence of subcutaneous tumor (0 points: absence; 1 point: presence); the presence of metastases in liver, lung, and lymph nodes (1 point per organ where metastasis was observed); and the presence of macrometastases (1 point per organ where a macrometastasis was observed). The maximum disease severity index was 7 points. For each mouse, the disease severity index was determined blindly by two independent observers who analyzed the tissues sections of lung, liver, and lymph nodes stained with HE.

### Statistical analysis

The Wilcoxon-Mann-Withney test was used to compare doubling time of cells, cell invasion and tumor growth. The log-rank test was used to compare survival between mice injected with cells expressing Mini5B and control cells. Pearson's chi-square test was used to compare the clinical score. Analyses were performed using StatXact 6.0 (Cytel Studio, Cambridge, MA) for exact nonparametric inference. A *p*-value of 0.05 or less was considered significant.

## Results

### Generation of stable clones

Our mini-mucin Mini5B was shown to be secreted efficiently as *N*-glycosylated and highly *O*-glycosylated molecules when transfected into COS-7 cells [13]. To test the function of Mini5B in breast cancer, we added an Ires–Luc tag to the Mini5B vector, and we prepared an empty control vector. A schematic representation of the two constructs used in this study is depicted in Fig. 1A. After transfection and G418 selection, four stable MCF7 clones from more than 48 screened clones for the control vector and two MCF7 stable clones from more than 70 screened clones for the Mini5B vector were obtained according to their luciferase activities (Fig. 1B). The Ires–Luc clone C5 and the Mini5B–Luc clone D6 were selected for further experiments. No morphological difference was observed under the microscope upon comparing Ires–Luc and Mini5B–Luc clones (data not shown). The two clones retained the same epithelial morphology and capability to form domes as the parental cell line. The luciferase activity was stable until at least 11 passages, and the luciferase activity was proportional to the number of cells over the range of dilutions tested (500 to 100 000 cells; data not shown). One Mini5B-Luc clone was chosen and sub-populations with empty shRNA or shRNA directed against the CYS sequence were derived (see after). Expression of the endogenous *MUC5B* was studied by qRT-PCR (TaqMan) and showed that the Ires-Luc clone and the Mini5B-Luc clone harbored a similar expression of *MUC5B* mRNA in comparison to the parental MCF7 cell line (Fig. 1C). Immunohistochemistry showed that Mini5B was expressed and secreted at the cell surface of the Mini5B–Luc clone chosen whereas no staining with the anti-MUC5B antibody was observed in the Ires–Luc clone and in the MCF7 parental cell line (Fig. 1D). Expression of the Mini5B transcript was evaluated from RNA extracted from two Mini5B clones and two Ires–Luc clones as negative controls. Expression of the transgene was detected by a PCR product of 159 bp (one Mini5B clone and one negative control clone shown; Fig. 1E). This shows that the expression of wild type MUC5B was very low in comparison to the expression of Mini5B. This was confirmed by western blotting with the anti-MUC5B antibody as no endogenous MUC5B was visualized but revealed a high molecular weight protein (≈250 kDa) for the Mini5B–Luc clone, whereas the predicted molecular weight for the non-glycosylated peptide is 71 kDa. No band was detected for the Ires–Luc clone (Fig. 1F).

### Inhibition of Mini5B expression in MCF7 cells by RNAi

To investigate further the putative role of Mini5B expression in tumor growth and metastasis, a Mini5B KD cell population was prepared by RNA interference from one chosen Mini5B clone (clone D6, Fig. 1B). The Mini5B–Luc clone was transfected with either a control shRNA (Mini5B–Luc empty shRNA) or an shRNA targeting the CYS sequence (Mini5B–Luc CYS shRNA). Inhibition of Mini5B production was quantified by Western blotting. Mini5B production was reduced by 96% in the CYS shRNA clone (Fig. 1F and 1G). No morphological difference was observed between the clones and shRNA cell populations obtained (data not shown).

### Mini5B promotion of MCF7 cell proliferation in vitro

The Mini5B–Luc clone and the control Ires–Luc clone were cultured for 7 days. Recombinant Mini5B decreased doubling time (18.6±1.4) in MCF7 cells in comparison with the Ires-Luc clone (26.2±4.0; Fig. 2A; *P* = 0.008) suggesting that Mini5B promotes cell proliferation.

### Invasive properties of MCF7 cells expressing Mini5B *in vitro*

The invasion of MCF7 cell clones and populations was evaluated using Matrigel assays. MCF7 cells are known to be poorly invasive *in vitro* [20]. This was confirmed in the Ires–Luc population, which had the lowest invasive ability, i.e., an invasiveness index <10% (Fig. 2B). Invasiveness was significantly greater in MCF7 cells expressing the Mini5B–Luc transgene (54.5%) compared with either the MCF7 control clone (7.6%; *P* = 0.006) or the cell population Mini5B–Luc carrying the shRNA directed against the transgene (30.8%; *P* = 0.029). This indicates an invasive effect of the mini-mucin Mini5B. The morphology of the invading cells stained with toluidine blue was similar to that of the cells before the Matrigel assay (Fig. 2C).

### *In vivo* promotion of tumor growth and metastasis by Mini5B

To investigate the contribution of MUC5B to the growth and dissemination of human breast cancer cells *in vivo*, SCID-Beige mice were injected s.c. with either the Mini5B–Luc MCF7 clone or Mini5B–Luc empty shRNA MCF7 cell population or Mini5B–Luc CYS shRNA MCF7 cell population or the Ires–Luc MCF7 clone. None of the mice showed any obvious clinical signs of tumor development during the first four weeks of observation following cancer cell injection. During the 118 days of the experiment, four mice were sacrificed. One mouse from the Ires–Luc group was sacrificed on day 100 because it had lost more than 20% of its body weight. This mouse displayed a small s.c. tumor but no metastasis was found in the lungs or liver. Two mice from the Mini5B–Luc group were sacrificed on days 23 and 86, and one mouse from the Mini5B–Luc empty shRNA group was sacrificed on day 23 because they displayed morbidities such as hunched posture, inactivity, and shortness of breath. The expression of Mini5B seemed to correlate with poor survival; e.g., 3/10 mice died during the course of the experiment in the two groups in which the Mini5B was expressed, whereas only 1/12 mice died in the two groups in which Mini5B was not expressed or in the KD group (*P* = 0.08). These first encouraging data suggested that Mini5B may increase MCF7 dissemination *in vivo*.

Inspection of the organs of the three mice expressing Mini5B sacrificed on days 23 and 86 revealed enlarged spleens and livers. The cellular organization of the liver of these three mice was disturbed and exhibited disseminated metastatic cells, as shown in Fig. 3A. HE staining also showed metastases in the lung and tracheobronchial lymph nodes (Fig. 3A). Immunohistochemistry showed that the metastatic cells were PCNA positive (Fig. 3B). Double immunostaining using anti-human E-cadherin [21] and anti-MUC5B showed that all E-cadherin positive cells expressed/secreted Mini5B as illustrated for thoracic lymph node metastasis (Fig. 3C).

Tumors were palpable on days 36 and 86 after injection in the Mini5B–Luc and Ires–Luc groups, respectively (Fig. 4A). At the end of the experiment (day 118), 4/5 mice in the Ires–Luc group, 4/4 mice in the Mini5B–Luc group, 1/3 mice in the Mini5B–Luc empty shRNA group, and 0/6 mice in the Mini5B–Luc CYS shRNA group displayed s.c. tumors. On day 118, the median volume of tumors was 33.1 mm^3^ for the Mini5B–Luc xenograft group, which was much greater than the tumor volume in the three other groups (≤0.5 mm^3^; *P*<0.02; Fig. 4A). By 8 weeks after s.c. injection, MCF7 cells expressing Mini5B–Luc had induced the development of s.c. tumors with a greater volume than the tumors observed in mice injected with MCF7 populations that did not express Mini5B. We conclude from these data that Mini5B stimulates tumor growth of MCF7 cells.

On day 118 after injection, mice were anesthetized and the tumors were visualized using the Xenogen luminescence apparatus. The bioluminescence signal was detected in all tumors. One mouse in the Mini5B–Luc group displayed a bioluminescence signal in the lung, showing the dissemination of cells expressing Mini5B (Fig. 4B). Using the Xenogen apparatus, we found that mice injected with Mini5B–Luc CYS shRNA displayed small s.c. tumors on day 118 but they were not palpable.

We analyzed Mini5B expression in s.c. tumors in each group and found that Mini5B was not detected by immunohistochemistry in both s.c. tumors of the Ires-Luc and Mini5B-Luc CYS shRNA groups while Mini5B was found secreted at the cell surface of s.c. tumors of Mini5B-Luc and Mini5B-Luc empty shRNA groups (Fig. 5A). These data confirmed that the MUC5B immunostaining visualized in tumors is due to the mini-mucin Mini5B and not to the endogenous mucin MUC5B.

On day 118, 2/5 mice in the Ires–Luc group and 4/4 mice in the Mini5B–Luc group displayed metastases in the liver, lungs, and thoracic lymph nodes (Fig. 5B and Table 1). The metastatic cells were PCNA (Fig. 5B) and E-cadherin (Fig. 5C) positive. On day 118, macrometastases (metastases which are >2 mm in greatest dimension) were observed in the lung in one mouse in the Ires–Luc group and in 4/4 mice in the Mini5B–Luc group (Fig. 5C and Table 1). By contrast, only one mouse in the Mini5B–Luc CYS shRNA group displayed metastasis in the liver (Table 1). The disease severity index was higher in mice injected with cells expressing Mini5B (Mini5B–Luc clones and Mini5B–Luc empty shRNA populations) than in mice injected with cells that did not express Mini5B (Ires–Luc and Mini5B–Luc CYS shRNA groups; *P* = 0.002) (Fig. 6). These preclinical data suggest that the mini-mucin Mini5B promotes tumor growth and cell dissemination in this model of s.c. xenografts.

## Discussion

Under physiological conditions, mucins play a protective role in epithelial tissues and are involved in the process of epithelial differentiation, growth regulation, modulation of cell adhesion, and cell signaling [22]. Numerous studies have now shown that abnormal mucin glycosylation is generally associated with a malignant transformation of epithelial cells [9], [23]. The overexpression of mucins observed in many cancers is important to the adhesion of and invasion by cancer cells, escape of cancer cells from the immune system, capture of biological molecules such as growth factors, and cellular growth [22].

Inappropriate or ectopic expression of mucins has been observed in some cancers. This is the case for MUC5B, which is expressed abnormally in gastric carcinomatous tissues and cell lines [24], and in lung adenocarcinomas [25], [26]. In mammary tissue, MUC5B protein is detected with a high frequency in breast cancer tissues, whereas the mucin is not expressed in normal control breast samples [10]. We have reported previously that all mammary tumors develop spontaneously in the transgenic MMTV-ras mouse strain produced Muc5b [27]. Among breast cancers, the luminal subtype accounts for 68% of all breast cancers and is associated with a high risk (70%) to develop metastases [28]. The MCF7 cell line, which express E-cadherin, estrogen receptor and progesterone receptor, is considered as a good model to mimic luminal breast cancer [29]. To understand further the implication of MUC5B in breast cancer pathogenesis, we transfected MCF7 cells with a recombinant mini-mucin MUC5B, and we evaluated the invasive and metastatic potential of these cells *in vitro* and in an s.c. xenograft model.

The predicted *MUC5B* transcript is 17.9 kb in length [5]–[7], [24] in agreement with Northern blot experiments [30]. The central exon of *MUC5B* is 10713 bp long and encodes a 3571 aa peptide made of seven CYS domains and the Ser/Thr/Pro-rich regions of the mucin, which carry numerous oligosaccharide chains. The large size of the peptide moiety of MUC5B (>5000 aa) led us to examine its role *in vitro* and *in vivo* using a mini-mucin made of a domain-specific region of 650 aa, which is representative of the central region of the full *O*-glycosylated mucin. This mini-mucin has previously been shown to be secreted as *N*-glycosylated and extensively *O*-glycosylated molecules when the vector is transfected in COS-7 cells [13].

We found that the mini-mucin promoted MCF7 cell proliferation *in vitro* and invasion in Matrigel. Xenograft experiments showed that the mini-mucin produced by MCF7 cells markedly affects tumor growth and is associated with aggressive tumor behavior. This finding is consistent with previous studies on the role of the other gel-forming mucin MUC2 in breast cancer [31]–[33]. One study showed that the colon cancer cell line LSLiM6 secretes MUC2, which, when inoculated into mice, induced metastasis in the liver [34]. Decreasing the expression of MUC2 by an antisense inhibition strategy reduced the ability of cells to colonize this organ.

Comparison of the frequency of tumor development and metastases between mice injected with the Ires-Luc clone and mice injected with the Mini5B-Ires-Luc CYS shRNA clone suggests that the better outcome was for the Mini5B-Ires-Luc CYS shRNA group. This is surprising as we expected similar results for these two groups. While the expression of the endogenous MUC5B seemed quite similar between clones and the parental cell line at the gene level and the protein level, we cannot totally rule out that expression of the wild type MUC5B which should not be inhibited in the Ires-Luc clone may be responsible in part for the differences observed.

The function of polymerizing mucins in the biology of breast cancer is unclear and the mechanisms by which mucin expression affects the tumorigenesis of breast cancer cells are poorly understood. The impact of mucin expression on cancer cell behavior may differ depending on the cell line and the mucin studied. It has been suggested that overexpression of MUC6 mucin domains inhibits invasion of cancer cells *in vitro* [35], whereas in some cases the expression of this secreted mucin seems to correlate with the degree of histopathology, which is related to malignant potential [36].

It is likely that the expression and abnormal glycosylation of our mini-mucin in MCF7 cells leads to modulation of cell–cell and cell–matrix interactions, facilitating the invasion of MCF7 cells. It has been shown using the two breast cancer cell lines MCF7 and T47D that MUC6 tandem repeats are good candidates for the high density of the common human cancer-associated Tn antigen found in these cell lines [37]. We can therefore suggest that overexpression of MUC5B tandem repeats in MCF7 cells may contribute to a higher expression of the Tn antigen. Abnormal glycosylation of MUC2 in LSLiM6 cells exposes Tn and sialyl Le^x^ antigens on the surface of tumor cells [34], and these glycan epitopes are important in adhesive interactions with the basement membrane and vascular endothelium by binding E-selectin, which favors metastasis [23], [34].

In conclusion, MUC5B overexpression in MCF7 cancer cells may stimulate aggressive behavior of tumor cells by increasing cell proliferation, tumor growth, and dissemination. Our study suggests that the development of vaccines directed against abnormally glycosylated MUC5B domains should be evaluated for breast cancer.
